# Slow wave synchrony during NREM sleep tracks cognitive impairment in prodromal Alzheimer's disease

**DOI:** 10.1002/alz.70247

**Published:** 2025-05-21

**Authors:** Omer Sharon, Vladislav Zhelezniakov, Yael Gat, Rotem Falach, Darya Narbayev, Tamara Shiner, Matthew P. Walker, Riva Tauman, Noa Bregman, Yuval Nir

**Affiliations:** ^1^ Center for Human Sleep Science, Department of Psychology University of California, Berkeley Berkeley California USA; ^2^ Helen Wills Neuroscience Institute University of California, Berkeley Berkeley California USA; ^3^ Department of Physiology & Pharmacology, Faculty of Medical and Health Sciences Tel Aviv University Tel Aviv Israel; ^4^ Sagol School of Neuroscience Tel Aviv University Tel Aviv Israel; ^5^ Cognitive Neurology Unit Tel Aviv Sourasky Medical Center Tel Aviv Israel; ^6^ Faculty of Medical and Health Sciences Tel Aviv University Tel Aviv Israel; ^7^ The Sieratzki‐Sagol Center for Sleep Medicine Tel Aviv Sourasky Medical Center Tel Aviv Israel; ^8^ Department of Biomedical Engineering, Faculty of Engineering Tel Aviv University Tel Aviv Israel; ^9^ Sagol Brain Institute Tel Aviv Sourasky Medical Center Tel Aviv Israel

**Keywords:** amnestic mild cognitive impairment, electroencephalography, memory, mild cognitive impairment, neurodegeneration, slow oscillation, slow wave

## Abstract

**INTRODUCTION:**

Alzheimer's disease (AD) disrupts human sleep architecture more severely than normal aging. However, it remains unclear how AD changes oscillatory neural activity during sleep, and whether such changes foreshadow cognitive decline in AD.

**METHODS:**

We used high‐density electroencephalography sleep recordings in 55 participants: (1) 21 healthy older adults, (2) 28 patients with amnestic mild cognitive impairment (aMCI)—a prodromal AD stage, and (3) 6 AD patients.

**RESULTS:**

Cognitive performance robustly decreases with the slow wave (SW) trough amplitude and its synchronization across broad frontocentral cortical areas. Thus, across the AD spectrum, slow wave synchrony declines with cognition, as in normal aging, but at an accelerated pace. Moreover, delayed rapid eye movement (REM) sleep onset in aMCI and AD patients was associated with deficient SW activity, suggesting insufficiently restorative non‐REM sleep.

**DISCUSSION:**

These findings suggest that impaired slow waves are closely linked to cognitive impairment and mark disrupted neural activity in AD progression.

**Highlights:**

- Detailed analysis of high‐density sleep electroencephalography was performed in amnestic mild cognitive impairment and Alzheimer's disease (AD) patients.
- Cognitive status robustly correlates with slow wave trough and its cortical spread.
- Delayed rapid eye movement sleep onset associated with AD correlates with diminished slow wave troughs.
- Impaired slow waves mark progressively disrupted neural activity in prodromal AD.

## 1 BACKGROUND

Sleep is essential to cognitive function including learning and memory.^1^, ^2^ Neurodegeneration observed in patients, animal models, and even atrophy in normal aging humans—is associated with both cognitive decline and sleep impairments.^3^, ^4^, ^5^ Alzheimer's disease (AD), the most frequent etiology of late‐life dementia,^6^ is a gradual multistep brain disorder that manifests first as mild cognitive impairments, frequently with episodic memory deficits, and gradually progresses into more severe cognitive decline and dementia, reflecting a massive neuronal degeneration, specifically excessive accumulation of amyloid beta (Aβ) and its formation into plaques, followed by hyperphosphorylation and breakdown of tau from the neural microtubule skeleton and their formation into neurofibrillary tangles. Degeneration is first observed in the medial temporal lobe in parallel to episodic memory impairment, and later in parietal and frontal brain areas, culminating in excessive neural loss.^7^, ^8^, ^9^

Amnestic mild cognitive impairment (aMCI) is considered a condition that often represents an early stage of AD. It is a syndrome defined by restricted memory decline while other cognitive domains remain relatively preserved. Many individuals with aMCI (≈ 60%–65%) eventually progress to dementia due to AD. aMCI diagnosis is associated with a 4‐fold increased risk for the development of AD dementia compared to healthy older adults,^10^ and a 2‐fold increase compared to individuals with non‐amnestic mid cognitive impairment (MCI).^11^ This view of aMCI as an early stage of AD is also supported by high levels of AD pathology in the brain of many aMCI patients *post mortem*,^12^, ^13^ in the cerebrospinal fluid,^14^ and using specific positron emission tomography tracer imaging.^15^, ^16^, ^17^

While there is abundant literature documenting significant sleep disturbances in AD patients^18^, ^19^ the nature and extent of sleep changes in aMCI compared to healthy aging remain less well understood and often controversial. For example, some studies report shorter non‐rapid eye movement (NREM) sleep in aMCI^11^, ^20^ while others do not.^21^ Although some sleep disturbances are likely present in individuals with aMCI,^11^, ^20^, ^21^, ^22^, ^23^, ^24^, ^25^, ^26^ a clear consensus has not been reached, with many studies reporting no significant differences. Importantly, it is still unclear whether these sleep disruptions are linked to memory impairment on an individual level.

We hypothesized sleep macro‐architecture (i.e., sleep stages) is not sensitive enough to detect associations between sleep and memory at the individual level in the early stages of disease (aMCI). Indeed, more detailed investigations of sleep oscillations have shown some potential in identifying deficits related to episodic memory impairment. Comparing healthy controls to MCI patients has revealed reductions in slow wave (< 4 Hz), theta (4–8 Hz), and sleep spindles power (12–16 Hz) during NREM sleep, with deficits related to episodic memory specifically.^20^, ^27^ Furthermore, lower fast spindle density across AD stages was confirmed using standard Mini‐Mental State Examination (MMSE) scores.^21^ However, findings remain inconsistent, as a newer study did not detect difference in sleep spindles between those with MCI and healthy controls.^28^ Additionally, a large‐scale analysis of sleep electroencephalography (EEG) data from 8044 participants replicated findings of reduced slow wave and spindle power in MCI and AD. However, when these features were used in a logistic regression model, they did not reliably distinguish MCI and AD patients from healthy controls,^29^ suggesting substantial individual variability. This underscores the need for novel sleep EEG markers and a more precise focus on specific clinical populations within the MCI subgroup.

In this study, we aimed to compare the sleep of aMCI patients to both healthy older individuals and AD patients, with the goal of identifying differences in brain activity during sleep (as measured by EEG oscillations) which could be reliably associated with individual cognitive performance. Our primary focus was comparing aMCI patients to healthy older adults while using a smaller sample of AD patients as a reference. This approach was chosen because aMCI represents a critical stage for potential intervention, given that cognitive symptoms have emerged, but significant neurodegeneration may still be mitigated. We used high‐density EEG (256 channels) and state‐of‐the‐art algorithms for detecting slow waves (SWs),^30^ the most robust electrophysiological hallmark of NREM sleep.

**RESEARCH IN CONTEXT**

1. **Systematic review**: A systematic literature search was carried out using common sources to identify studies investigating sleep of patients in the early stages of Alzheimer's disease (AD). Only a few studies have focused specifically on patients with amnestic mild cognitive impairment, and it remains unclear whether sleep disruption is linked to memory impairment in this population.
2. **Interpretation**: Our findings indicate that impairments in synchronizing large neural populations into slow waves during non‐rapid eye movement sleep may play a pivotal role in dementia due to AD. This dysfunction might inform the level of disrupted neural activity due to AD progression.
3. **Future directions**: Future studies may investigate a causal association among impaired slow waves, cognitive symptoms, and AD pathology using longitudinal studies to determine whether this impairment further contributes to disease progression. Additionally, imaging and additional biomarkers could determine whether impaired slow waves in patients are related to accumulation of tau or neurodegeneration.

## 2 METHODS

### 2.1 Participants

The study was approved by the medical institutional review board at the TASMC (Tel Aviv Sourasky Medical Center). All participants provided written informed consent. The cohort included a total of 66 participants. Of them, 30 were aMCI patients, 29 were healthy age‐matched controls, and 7 were AD patients (see Table 1 for summary after exclusions). Patients were recruited from the Memory and Attention Disorders Center, Neurology Department, TASMC, Israel as follows: individuals with subjective cognitive complaints were referred to the clinic's general practitioners, neurologists, geriatricians, and psychiatrists. Those diagnosed with aMCI or AD were then invited to participate in the sleep study. Healthy volunteers were recruited from the community while those with low cognitive scores (Montreal Cognitive Assessment [MoCA] < 26, *n* = 5) were excluded from further analysis (Figure S1 in supporting information). Exclusion criteria included any history of sleep apnea, psychiatric disorders, neurological disorders, stroke, or head injury; current alcohol or drug misuse; trans‐meridian travel within the last week; and use of hypnotics (one unimpaired participant was excluded due to an inability to fall asleep during the lab session, resulting in no sleep data for analysis). Patients with moderate or severe sleep apnea (apnea–hypopnea index [AHI] > 15) were excluded from further analysis (*N* = 5; 1 AD, 2 aMCI, and 2 healthy participants), see below. In total, 55 participants were included in the analysis, 21 healthy older adults, 28 aMCI patients, and 6 AD patients.

**TABLE 1:** Demographics and cognitive performance of included participants.

	Control (*N* = 21) mean (SD)	MCI (*N* = 28) mean (SD)	AD (*N* = 6) mean (SD)	Control vs. MCI	Control vs. AD	MCI vs. AD
**Demographics**						
Age (years)	67.09 (5.77)	71.35 (6.46)	69.83 (8.79)	0.03	n.s	n.s
Male sex (*n*;%)	10 (47.6%)	15 (53.8%)	5 (83%)	n.s	n.s	n.s
Right‐handed (%)	20 (93%)	25 (89%)	6 (100%)	n.s	n.s	n.s
**Cognition**						
MMSE	29.39 (1.14)	26.59 (2.62)	22.16 (4.49)	<10^−4^	<10^−3^	0.02
MoCA	27.90 (1.51)	22.04 (3.01)	18.16 (5.45)	10^−8^	0.001	0.05
MoCA free recall	3.66 (1.31)	1.07 (1.59)	1.33 (1.86)	10^−4^	0.008	n.s
d‐prime	1.92 (0.72)	0.96 (1.07)	0.15(0.86)	0.002	0.009	n.s

*Notes*: Values are presented as mean (SD) unless otherwise noted. Comparisons between groups reflect *p* values. d‐prime, sensitivity index from the overnight recall test.

Abbreviations: AD, Alzheimer's disease; MCI, mild cognitive impairment; MMSE, Mini‐Mental State Examination; MoCA, Montreal Cognitive Assessment; SD, standard deviation.

### 2.2 Experimental procedure

#### 2.2.1 Cognitive testing

Participants were invited to the lab 3 hours before their usual bedtime. After signing informed consent, a cognitive assessment was conducted using MMSE and MoCA.^31^ Then, participants completed a movie‐viewing paradigm using 120 short (< 26 seconds) black‐and‐white YouTube video clips depicting real‐world scenes with naturalistic narrative content.^32^ Each participant watched 100 movies before sleep in a pseudorandom order. In the morning, participants had an additional viewing session while 80 movies were repeated and 20 were new. After each movie participants were asked “Have you seen this movie before?” (“yes” or “no”). The sensitivity index *d'* was calculated using the difference between the inverse of the cumulative distribution function of the hit rate and the false alarm rate.

#### 2.2.2 Polysomnographic recordings

High‐density polysomnography (PSG) was recorded throughout the night using a 256‐channel recording system (Electrical Geodesics, Inc. [EGI]). Each carbon‐fiber electrode, consisting of a silver chloride carbon fiber pellet, a lead wire, and a gold‐plated pin, was injected with conductive gel (Electro‐Cap International). Signals were referenced to Cz, amplified via an AC‐coupled high‐input impedance amplifier with an antialiasing analog filter (NetAmps 300, EGI), and digitized at 1000 Hz. Electrode impedance was verified to be < 50 kΩ. Respiratory monitoring included blood saturation with SpO2 sensor (Nonin 8000J), nostril airflow, and respiratory effort with chest and abdomen belts (1399/Piezo Crystal Effort Sensor, SleepSense) and was completed in most (49/66) participants.[Fig alz70247-fig-0001]

### 2.3 Data analysis

#### 2.3.1 Clinical sleep assessment

Offline respiratory assessment was performed by a sleep clinician (R.T.) certified by the American Academy of Sleep Medicine (AASM). The AHI was obtained when possible, otherwise a binary score was derived. Subjects diagnosed with breathing issues (moderate to severe obstructive sleep apnea [OSA; AHI ≥ 15]), central sleep apnea, or hypoventilation were ruled out and excluded from further analysis (*N* = 5; 1 AD, 2 aMCI, and 2 healthy participants). While a 7% exclusion rate (5/66) may seem low compared to typical prevalence in older adults, this is expected because participants with a known history of sleep apnea were excluded before arriving at the lab. Although OSA has been linked to neurodegeneration,^33^ recent large‐scale analyses suggest its prevalence is similar across healthy controls, MCI, and AD patients.^29^ Given that our study focused on neural activity during sleep rather than sleep disruption from apnea, we prioritized an apnea‐free sample to ensure clearer interpretations.

#### 2.3.2 Sleep scoring

**FIGURE 1 alz70247-fig-0001:**
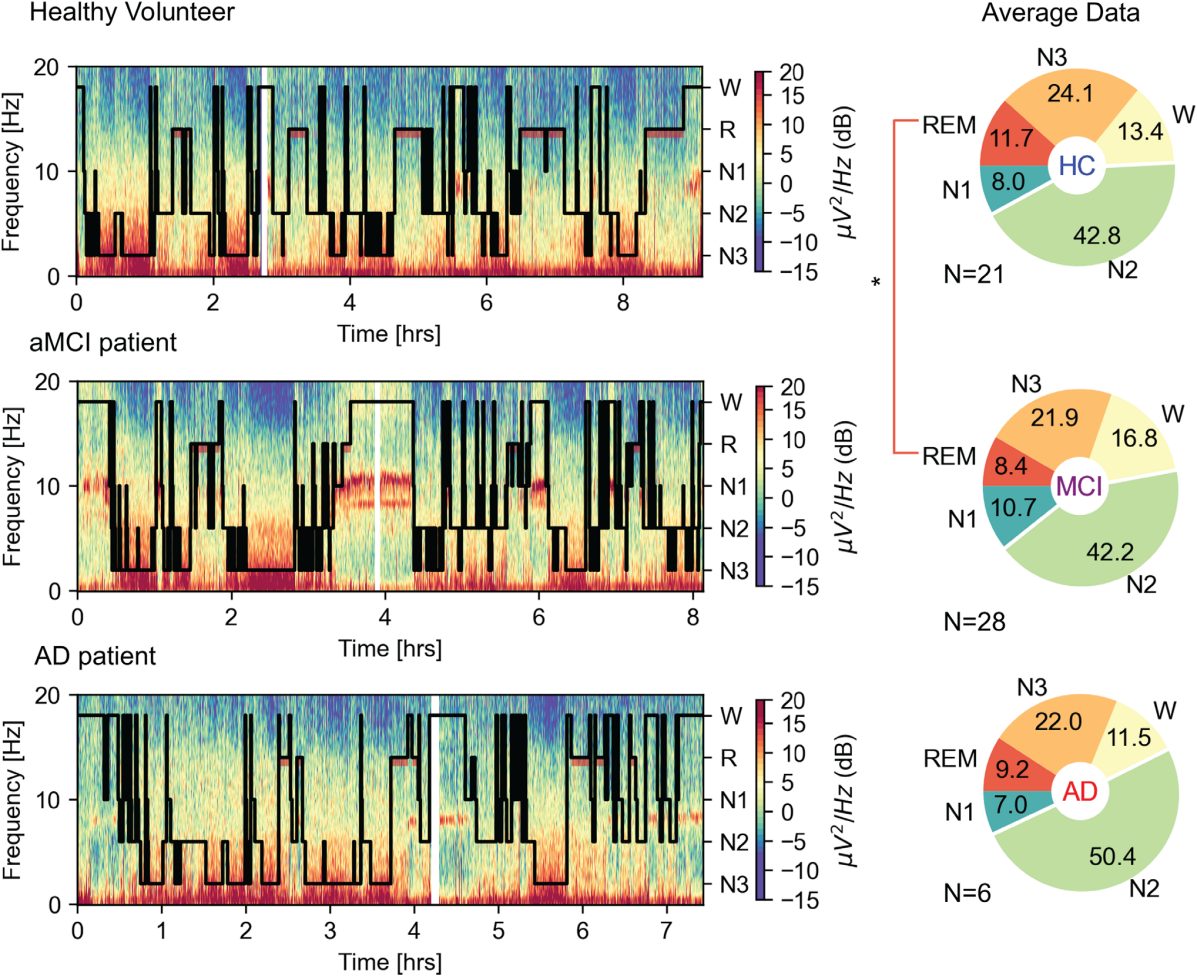
Representative hypnograms and spectrograms of healthy, aMCI, AD, and average sleep composition. Left, Spectrogram depicting EEG power over Pz (referenced to linked mastoids) across the whole night for three representative participants, a healthy volunteer (upper panel), an aMCI patient (middle panel), and an AD patient (bottom)—all overlaid with sleep scoring classification according to American Academy of Sleep Medicine (see Methods section). Right, Significant differences between the groups were observed only in %N1 and % REM. AD, Alzheimer's disease; aMCI, amnestic mild cognitive impairment; EEG, electroencephalography; REM, rapid eye movement.

Sleep scoring was performed according to AASM guidelines^34^ using the sleep module in the Visbrain Python package^35^ and the SleepEEGPy platform.^36^ To this end, EEG data from F3/F4, C3/C4, and O1/O2 was re‐referenced to the contralateral mastoid, resampled to 250 Hz, and visualized in 30 second epochs together with synchronized electrooculograms and electromyogram from submental electrodes. Scoring was further verified by inspecting the spectrogram of the Pz electrode with the hypnogram overlaid (Figure 1).

#### 2.3.3 EEG analysis

EEG was preprocessed using the MNE Python package.^37^ Because the analysis focused on SWs, we focused on sleep stages in which slow wave activity is more prevalent. Specifcally, we extracted EEG segments scored as either N2 or N3 sleep from all subjects for further analysis. Continuous data were bandpass filtered between 0.5 and 40 Hz using a finite impulse response (FIR) filter, and downsampled to 250 Hz. Artifacts were excluded based using 5 second epochs by identifying data exceeding the 400 µv threshold and epoch windows or whole channels using an iterative process that minimizes the amount of discarded data^38^ (and see code).

Individual SWs were detected in the range of 0.3 to 1.5 Hz (sometimes termed slow oscillations^39^) using established algorithms^40^, ^41^ as implemented in the YASA Python package, using default standard parameters.^42^ In short, each electrode was band‐pass filtered between 0.3 and 1.5 using a FIR filter with a transition band of 0.2 Hz. Negative peaks between –40 and –200 µv were detected and positive peaks between 10 and 150 µv. For each slow wave trough, the nearest positive peak was found and the peak‐to‐peak amplitude was computed. In addition, the duration of the negative phase and positive phase was computed. The negative phase is the time between the first zero crossing (before the trough) and the second zero crossing (between the peaks) and the positive peak is the time between the zero crossing between the peaks and the last zero crossing (after the positive peak). Waves were included only if peak‐to‐peak amplitude was within 75 to 300 µv; the negative phase was between 0.1 and 1.5 seconds and the positive peak was between 0.1 and 1 seconds. When conducting the entire scalp analysis, the electrodes of a particular participant were included if at least 25 SWs were detected. Analysis of cortical involvement has been previously described^30^ and adjusted here for 256 channels by excluding SWs observed only in one to two channels.

#### 2.3.4 Statistics

All statistical comparisons were carried out using the Pingouin Python package for statistical analysis.^43^ All correlations reported are Pearson correlations unless stated otherwise. Direct comparisons were carried out using a *t* test when both populations were normally distributed (verified with Shapiro–Wilk test) and using a Mann–Whitney test otherwise, and then a *U* value is reported instead of a *t* value. The criteria for significance was 0.05 two sided unless stated otherwise. Multiple comparisons correction was performed using the Benjamini–Hochberg method for false discovery rate (FDR)^44^ as implemented in the Python stats models package.^45^ Comparing 256 channels SW trough across groups was performed using the MNE cluster permutation test.^46^ A Fisher test^47^ was used to compare two regression coefficients to each other.

## 3 RESULTS

### 3.1 Memory performance across the AD spectrum

Patients with aMCI and AD were referred from the Cognitive Neurology Unit at TASMC for an overnight sleep‐lab session. Age‐matched healthy volunteers were recruited from the community (age range: 52–85, Table 1). Cognitive performance was evaluated using standard questionnaires including the MMSE and the MoCA, and using an overnight memory consolidation paradigm quantifying morning recollection of stimuli presented in the pre‐sleep evening session (see Methods section). Cognitive performance was progressively poorer across the AD spectrum, as expected (Table 1). Scores on the MoCA test were lowest in AD patients (18.16 ± 5.45), significantly lower than those of aMCI patients (22.25 ± 3.15, *U* = 126, *p* = 0.05), which were significantly lower than those of healthy controls (27.90 ± 1.51, *U* = 559, *p* < 10^−8^). Focusing on the memory questions of the MoCA, healthy controls recalled 3.66 ± 1.32 words on average (out of 5), while aMCI and AD patients recalled 1.21 ± 1.72 words (*U* = 503, *p* ≤ 10^−4^) and 1.33 ± 1.86 words (1.33 ± 1.86, *U* = 72, *p* = 0.58), respectively. Similarly, MMSE scores of AD patients were minimal (22.16 ± 4.49) and significantly lower than aMCI patients (26.64 ± 2.58, *U* = 135, *p* = 0.02), and these were significantly lower than those of healthy volunteers (29.38 ± 1.14, *U* = 432, *p* < 10^−4^). Patients also performed worse on the overnight memory test, as quantified using a composite *d'* score encompassing the number of hits, false alarms, correct rejections, and misses (see Methods section). Accordingly, overnight memory accuracy *d'* in healthy volunteers averaged 1.92 ± 0.72, significantly higher than both aMCI (average of 0.97 ± 1.05, *U* = 354, *p* = 0.002) and AD patients (average of 0.16 ± 0.86, *U* = 67, *p* = 0.009, Table 1).

### 3.2 Changes in sleep architecture with cognitive decline and neurodegeneration

We recorded high‐density EEG and overnight polysomnography and performed sleep scoring according to AASM guidelines (Methods). Overall, sleep architecture was quite stable across the AD spectrum (Figure 1). The only significant differences between healthy controls and aMCI patients’ sleep architecture were in regard to REM sleep, occurring 54.6 minutes later in the night (95% confidence interval [CI] = 14.5, 94.7, *U* = 392, *p* = 0.004) summing to a total of 17.2 minutes’ reduction (32% compared to healthy controls, 95% CI = –34.6, 0.2) in the amount of REM sleep (*U* = 192, *p* = 0.04, Figure 1). No significant differences were observed when comparing aMCI and AD patients and no significant differences emerged when comparing healthy controls and AD patients (Table S1 in supporting information), possibly due to small AD patient sample.[Fig alz70247-fig-0002]

Additionally, none of the sleep architecture parameters correlated with cognitive decline as measured by the MoCA test, except REM sleep latency (*N* = 55, *r* = –0.40, 95% CI = –0.61, = 0.15, *p* = 0.003) and N3 sleep latency representing the onset of deep slow wave sleep (*N* = 55, *r* = –0.31, 95% CI = –0.53, 0.05, *p* = 0.02). Accordingly, individuals who entered REM and N3 sleep later exhibited worse cognitive performance (Table S2 in supporting information). Taken together, this suggests that sleep architecture is only modestly associated with cognitive performance across the AD spectrum and that revealing relevant changes requires detailed analysis of EEG recordings beyond changes in sleep architecture (see Discussion section).

### 3.3 Lower slow wave trough amplitude correlates with poorer cognitive performance

**FIGURE 2 alz70247-fig-0002:**
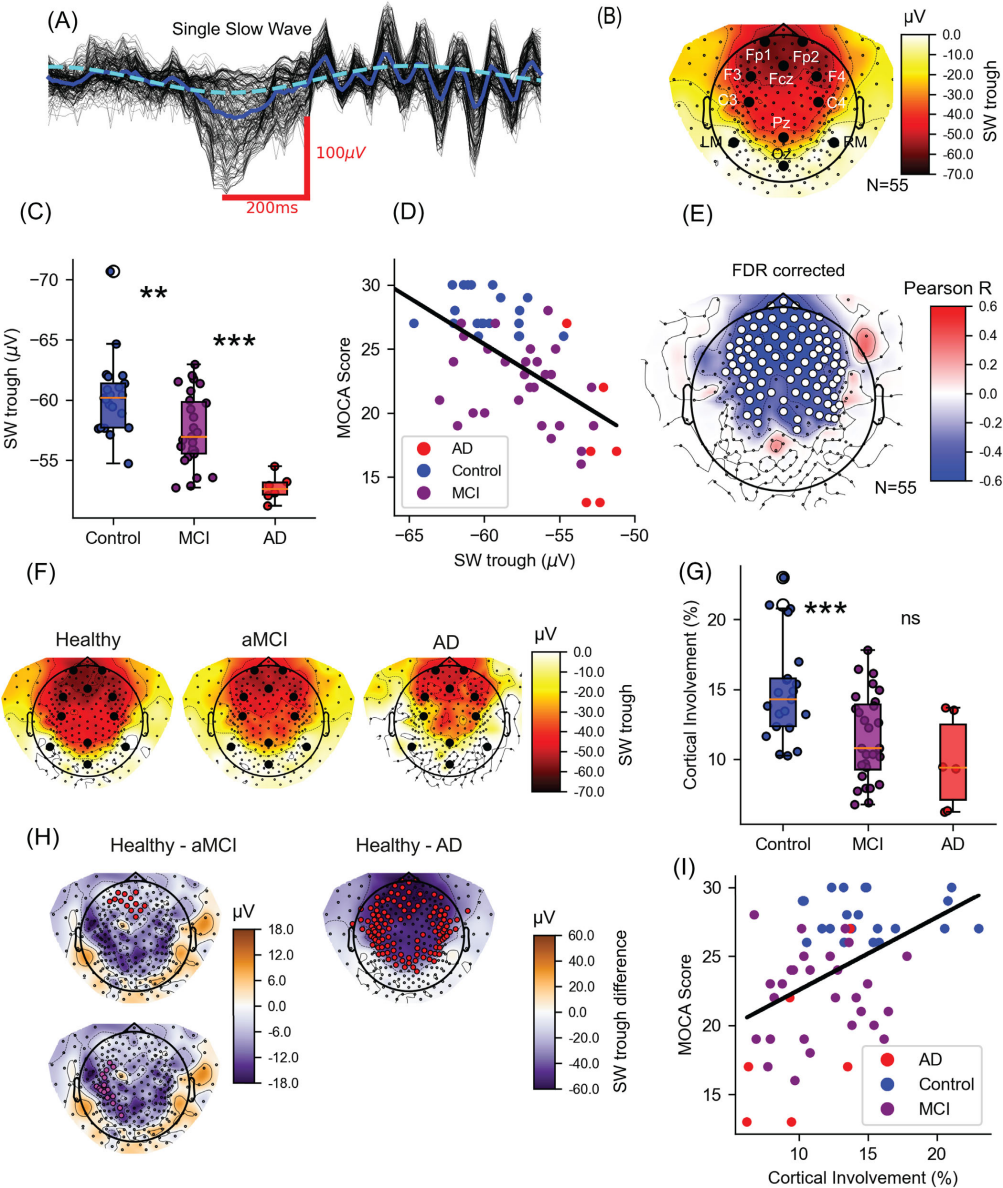
NREM sleep slow‐wave (SW) trough amplitude and its relation to cognitive decline. A, Example of a single SW, depicting the trough of SWs across all clean channels of a single individual (black), the average channel (blue), and its later filtered version (cyan) that is later analyzed on a single subject, single electrode, and population basis. B, Average SW amplitude across all participants included in the study (*N* = 55)—note the typical frontal topography. C, Comparison of the negative SW amplitude among healthy controls (blue), aMCI patients (purple), and AD patients (red) in a frontal ROI, the inset shows the topographical distribution of the SW trough across the scalp. D, The negative SW amplitude of individual participants in a frontal ROI of robustly correlated to cognitive performance as measured by MoCA scores. E, Pearson correlation of negative SW amplitude and MoCA score across all electrodes in the montage; white dots indicate significant correlation after FDR correction for multiple comparisons. F, Average topography of SW trough for healthy older adults (left), aMCI patients (center), and AD patients (right)—note the difference in SW spread. G, Comparison of cortical involvement in SWs across the entire scalp between healthy controls (blue), aMCI patients (purple), and AD patients (red) in a frontal ROI. Cortical involvement in SW was in particular sensitive to the difference between healthy older adults and aMCI patients. H, Topography of the difference in SW trough amplitude between healthy older adults and aMCI patients, significant channels in a cluster permutation are marked in red for the first cluster (above) and magenta for the second (bottom), at the right side is the same figure comparing healthy older adults to AD patients. I, Cortical involvement level of individual participants correlated with cognitive performance as measured by MOCA scores. AD, Alzheimer's disease; FDR, false discovery rate; MoCA, Montreal Cognitive Assessment; NREM, non‐rapid eye movement; ROI, region of interest; SW, slow wave.

Next, we went beyond sleep scoring and proceeded to analyze in detail the properties of SWs occurring in artifact‐free NREM (N2,N3) sleep (Methods). For each participant separately, SW amplitude was derived from the detected SW (Figure 2A, Methods) in a frontal region of interest (ROI) comprising 52/256 electrodes where SW amplitude is maximal (Figure S2A in supporting information). Participants showed the typical^5^ frontal SW scalp distribution (Figure 2B). Healthy controls had a median negative SW trough of –60.18 ± 3.67µV while aMCI patients showed a significantly smaller trough of –56.95 ± 4.27µV (*U* = 148, *p* = 0.003). AD patients showed the smallest SW trough of –52.61 ± 1.01µV and were significantly smaller compared to both aMCI patients (*U* = 228, *p* < 10^−4^) and healthy controls (*U* = 126, *p* < 10^−5^, Figure 2C). Across the entire scalp, we saw continuously degraded SW troughs both in amplitude and cortical spread (Figure 2F). Healthy older adults showed two significant clusters, one of frontal electrodes showing a larger SW trough compared to aMCI (Figure 2H left, red electrodes, permutation test *p* = 0.027) and a second cluster extending to parietal areas in the left hemisphere (Figure 2H left, magenta electrodes, permutation test *p* = 0.028). Comparing healthy controls and AD patients showed one large cluster (Figure 2H right, red electrodes, *p* = 0.001). In line with previous reports,^48^, ^49^ SW trough was significantly lower for males compared to females (–56.49 ± 3.03 µV, –59.89 ± 3.60 µV [± SD], *t* = 3.73, *p* = 0.0005).

Next, we sought to test whether the link between cognitive performance and SW trough can go beyond the group label to indicate cognitive performance at the individual level. We found a robust correlation between the SW trough amplitude and MoCA score (*N* = 55, *r* = –0.60, 95% CI = –0.74, –0.4, *p* = 10^−6^; Figure 2D) while age showed no correlation (*N* = 55, *r* = 0.11, *p* = 0.42, Figure S2D). These results remained significant when excluding AD patients (*N* = 49, *r* = –050, 95% CI = –0.69, –0.26, *p* = 2*10^−4^), when excluding healthy controls (*N* = 34, *r* = –0.47, 95% CI = –0.7, –0.15, *p* = 0.005), and trended toward significance even when inspecting only aMCI patients (*N* = 28, *r* = –0.34, 95% CI = –0.63, –0.04, *p* = 0.07). This association was weaker for the amplitude of the positive SW peak (*N* = 55, *r* = 0.28, 95% CI = 0.02, 0.51, *p* = 0.03, Fisher *Z* = 2.02, *p* = 0.04) and intermediate in strength when peak‐to‐peak amplitude was used (*N* = 55, *r* = 0.45, 95% CI = 0.22, 0.65, *p* = 0.0004). Finally, we computed the correlation between MoCA and SW trough amplitude across all electrodes. After correcting for multiple comparisons using FDR (Methods), a robust correlation was revealed across a wide area over the frontal and central cortices (Figure 2E), indicating that the results are robust above and beyond the precise ROI choice.

To test whether other features of SWs also correlated with individual cognitive performance, we compared SW density (the number of detected SWs per unit time). Healthy controls did not differ from aMCI patients, while AD patients showed approximately one third of the amount of SWs per minute (Figure S3A in supporting information). At the individual level, despite a robust correlation between SW density and SW trough amplitude (Figure S3B; *N* = 54, *r* = –0.47, 95% CI = –0.66, –0.24, *p* = 0.0003), SW density was not correlated with cognitive performance (*N* = 54, *r* = 0.19, 95% CI = –0.07, 0.44, *p* = 0.15, Figure S3C).

Given the relation between SW amplitude at single electrodes and the degree of cortical involvement,^50^ that is, synchronized SWs across larger cortical regions, we tested whether cortical involvement may also be related to cognitive performance, as recently demonstrated in the context of tau accumulation in healthy aging.^30^ Indeed, we found a robust correlation between cortical involvement and MoCA scores (*N* = 55, *r* = 0.45, 95% CI = 0.21, 0.64, *p* = 0.0005, Figure 2I). On average, cortical involvement was lower in aMCI patients (11.54 ± 3.13%) compared to healthy individuals (14.97 ± 3.68, *t* = 3.43, *p* = 0.001) while AD patients showed a lower but not significantly different cortical involvement level of 9.75 ± 3.29% compared to aMCI (*t* = –1.21, *p* = 0.26, Figure 2G). No correlation with age was observed (*N* = 55, *r* = –0.14, 95% CI = –0.4, 0.12, *p* = 0.28, Figure S2E) and results remain robust in multiple regression including age and sex (*N* = 55, β = –0.73, *t *= –4.8, *p* = 10^−5^, *r* ^2 ^= 0.38; age and sex were not significant in determining MOCA). Results remained robust (*N* = 40, *r* = –0.65, 95% CI = –0.8, –0.43, *p* < 10^−5^) even when excluding participants with undetermined sleep breathing performance (*N* = 15, due to technical issues, see Methods).

Beyond MoCA score, we found that the ability of participants to identify the movies in the overnight consolidation test (Methods) showed a similar pattern, whereby the slow wave trough was predictive of *d'* score reflecting accurate recollection (*N* = 49, β = –0.12, *t* = –0.2.6, *p* = 0.01, *r* ^2^ = 0.20). Age was also a significant predictor for accurate recollection (β = –0.05, *t *= –2.14, *p* = 0.03). But both of these associations were not significant when excluding the AD patients.

### 3.4 Insufficiently restorative NREM sleep is associated with delayed REM sleep onset

**FIGURE 3:**
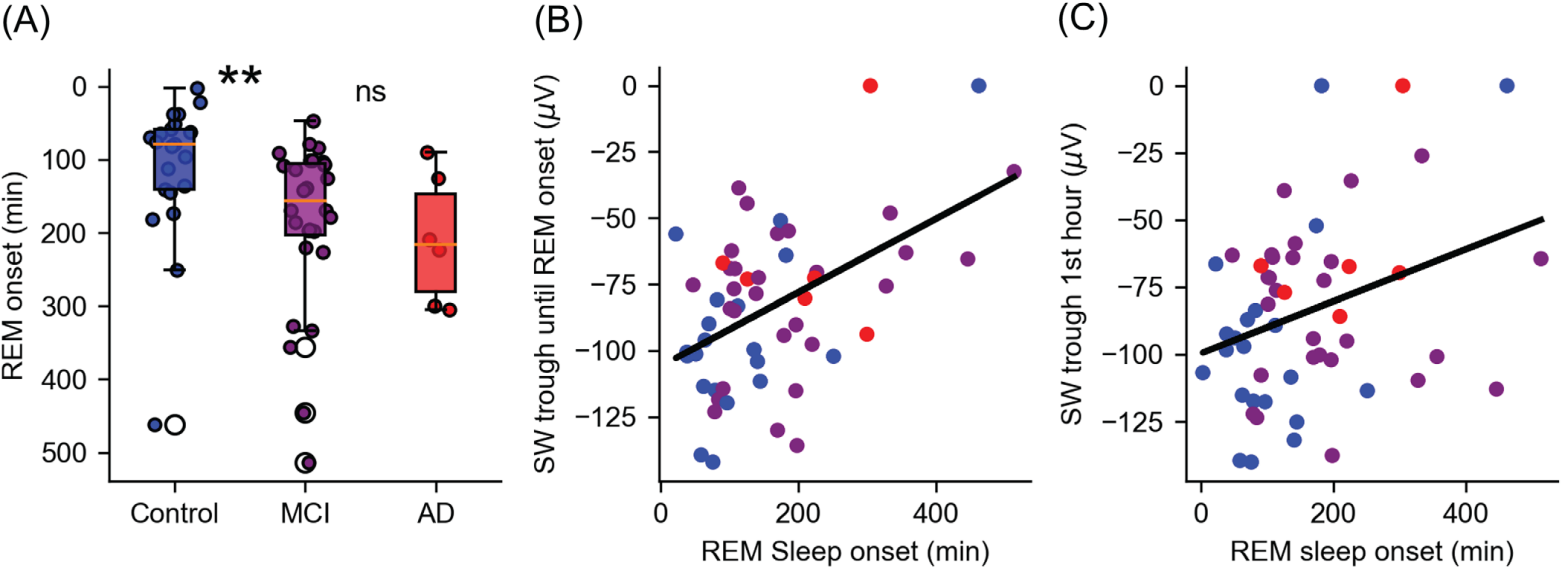
Relationship between slow wave decline and late REM sleep onset. A, Average REM onset (latency) in minutes in healthy controls (blue), aMCI patients (purple), and AD patients (red). B, Low SW trough before REM sleep onset was correlated with the delayed transition to REM sleep. C, Low SW through the first hour of NREM sleep was correlated with the delayed transition to REM. AD, Alzheimer's disease; aMCI, amnestic mild cognitive impairment; NREM, non‐rapid eye movement; REM, rapid eye movement; SW, slow wave.

The two main observations regarding sleep changes in early aMCI were (1) delayed REM sleep onset (Table 1, Figure 3A), and (2) lower SW trough amplitude (Figure 2). We hypothesized that these two observations may be related. Lower SW amplitude may represent suboptimal NREM sleep that is insufficiently restorative and therefore transition to REM is delayed. We tested this by focusing analysis on SW trough amplitude in the initial NREM episode, before the occurrence of any REM sleep. In line with the hypothesis, we found that SW trough amplitude during NREM sleep preceding REM onset was positively correlated with REM sleep onset (*N* = 54, *r* = 0.49, 95% CI = 0.26, 0.68, *p* = 0.0001, Figure 3B). Similar results were observed when focusing on the first NREM hour (*N* = 54, *r* = 0.32, 95% CI = 0.06, 0.54, *p* = 0.01, Figure 3C). However, beyond the initial REM sleep epoch, no significant relationship was observed for SW trough amplitude in intervals of NREM sleep occurring between REM episodes and the timing of the subsequent REM sleep interval.

## 4 DISCUSSION

The synchrony of NREM SWs tracks AD progression, from MCI to severe dementia (Figure 2A). Analysis shows a robust correlation between SW trough amplitude and MoCA scores, independent of age and sex. Importantly, this correlation was not due to differences between severe AD patients and healthy controls. Rather, it was observed within the aMCI population itself, across varying levels of cognitive decline. This association extended to a broad set of frontal and central cortical areas (Figure 2E). Additionally, impaired SW synchrony also manifested at a larger scale as reduced SW cortical involvement in aMCI, which was also strongly associated with cognitive performance. An association between individual aMCI memory performance and specific electrophysiological features of sleep oscillations has not been previously demonstrated. The lower sensitivity of peak‐to‐peak and insensitivity of SW density (Figure S3) may explain why prior studies could not detect such associations with cognitive performance.^11^, ^22^, ^26^ In addition, recruitment for this cohort focused on aMCI patients, reducing the heterogeneity in MCI samples.

The findings suggest that the impact of AD on sleep operates along a continuum, as the effects of AD pathology in healthy aging.^30^, ^51^, ^52^ However, in AD, the decline in SW integrity rapidly accelerates in parallel with cognitive decline. What mechanism could drive the reduction in SW amplitude across the AD spectrum? The SW trough amplitude in scalp EEG measures the synchronous transition of large neuronal populations from a silent OFF state to an active ON state of synaptic transmission and action potential firing.^50^, ^53^ In this context, the reduced amplitude may indicate cases in which some brain atrophy already challenges small neural populations in resuming their activity,^54^ or may index tau pathology preventing neural populations from effectively synchronizing their activities.^30^, ^55^ Future studies combining sleep recordings in aMCI with measurements of cortical thickness and tau spread could help distinguish between these possibilities.

What are the functional consequences of degraded SWs? The data show that reduced SW amplitude correlates with delayed REM sleep onset suggesting that, in some individuals, NREM is insufficiently restorative and may delay the transition into REM sleep (Figure 3C,E). A recent study reported EEG slowing during REM sleep in aMCI patients^56^ potentially reflecting disrupted SW homeostasis that could also result in SW invasion into REM sleep. These findings suggest that degraded SWs could affect additional aspects of sleep and their corresponding functions. Moreover, degraded SW may impair cortical–hippocampal synchrony, associated with memory consolidation,^39^ and through neurovascular coupling, could reduce the efficiency of brain waste clearance, potentially accelerating AD pathology.^57^

This study has several limitations. First, it is a cross‐sectional study and cannot pinpoint the causal role of SWs during sleep in cognitive decline. Future studies could extend the findings by testing whether degraded SWs causally accelerate cognitive decline across the AD spectrum and other dementias. These efforts could include longitudinal tracking of sleep EEG and AD pathology from healthy aging to dementia, investigating sleep EEG responses to novel drug therapies or other non‐pharmacological interventions, and exploring the specificity of these results to the AD spectrum versus other neurodegenerative disorders. Further, this study relied on global cognitive measures rather than a detailed neuropsychological battery. While these measures provide a useful overview of cognitive performance, a more comprehensive assessment could help better characterize the specific cognitive domains affected by sleep impairment in patients. Last, the study was focused on aMCI patients whereas the AD patient group was a small reference group, limiting the ability to draw definitive conclusions about differences between aMCI and AD patients. Future studies with larger number of AD patients are needed to better characterize sleep‐related changes between aMCI and AD patients specifically.

Clinical diagnosis and screening for prodromal AD remain challenging.^58^ Although biomarkers of AD pathology are increasingly available, confirmation of AD pathology can be inconclusive, unless the disease has already progressed significantly. This study suggests that reduced SW synchrony may offer an additional, direct marker of impaired neural activity at the earliest stage of early AD progression.

## AUTHOR CONTRIBUTIONS

Yuval Nir conceived the study and secured funding; Noa Bregman led patient recruitment and clinical diagnosis together with Tamara Shiner; Omer Sharon designed tasks; Omer Sharon, Vladislav Zhelezniakov, Yael Gat, Rotem Falach performed data collection; Vladislav Zhelezniakov and Omer Sharon analyzed the data; Riva Tauman, Vladislav Zhelezniakov, and Yael Gat led sleep breathing assessment; Omer Sharon and Yuval Nir wrote the manuscript. All authors provided feedback and commented on the manuscript.

## CONFLICT OF INTEREST STATEMENT

Nothing to declare.

## CONSENT STATEMENT

The study was approved by the medical institutional review board (IRB) at the TASMC. All participants provided written informed consent.

## Supporting information



Supporting Information

Supporting Information
