# Supplementary material for: Slow wave synchrony during NREM sleep tracks cognitive impairment in prodromal Alzheimer's disease
Source: Alzheimers Dement. 2025 May 21;21(5):e70247. doi: 10.1002/alz.70247 (PMC12094885; doi:10.1002/alz.70247)
Supplement: Supplementary file 1 — Supporting Information [file ALZ-21-e70247-s001.docx]

## Supplementary Materials

**Figure S1: Flow chart of participant recruitment**


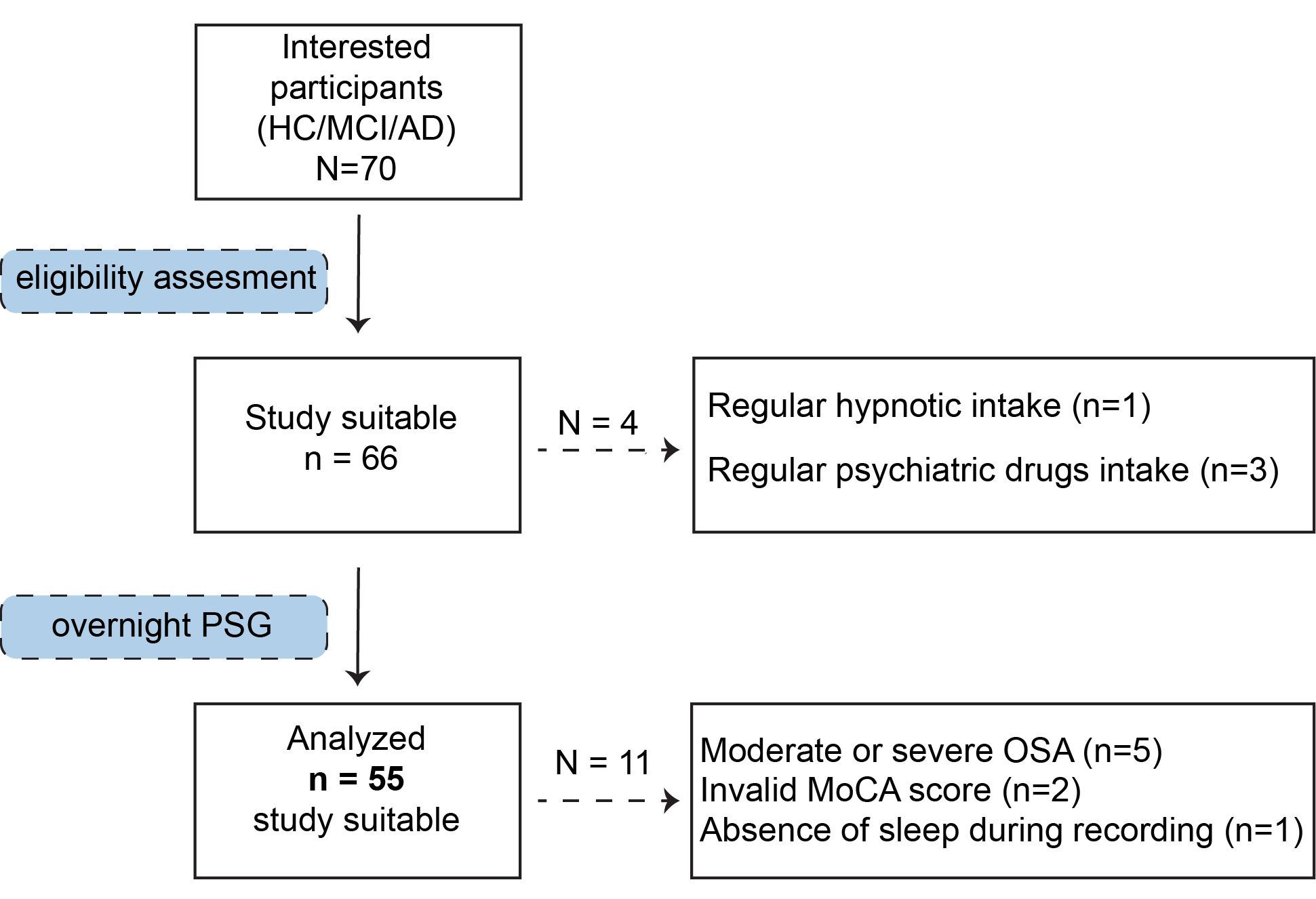


**Table S1: Sleep architecture and its relation to cognitive status**

| Sleep Architecture direct comparisons | | | | | | |
| --- | --- | --- | --- | --- | --- | --- |
|  | MCI patients vs Healthy Control | | MCI vs AD patients | | Healthy controls vs AD patients | |
|  | U-val | p-val | U-val | p-val | U-val | p-val |
| **TIB** | 253 | 0.413 | 83 | 0.982 | 70 | 0.711 |
| **SPT** | 257 | 0.461 | 79 | 0.839 | 70 | 0.711 |
| **WASO** | 320 | 0.599 | 101 | 0.466 | 72 | 0.629 |
| **TST** | 237 | 0.254 | 72 | 0.619 | 69 | 0.754 |
| **N1** | 318 | 0.635 | 101 | 0.456 | 75 | 0.502 |
| **N2** | 265 | 0.572 | 62 | 0.331 | 51 | 0.502 |
| **N3** | 201 | 0.063 | 74 | 0.684 | 76 | 0.465 |
| **REM** | 192 | 0.040 | 74 | 0.684 | 87 | 0.175 |
| **NREM** | 253 | 0.413 | 79 | 0.843 | 66 | 0.884 |
| **SOL** | 226 | 0.176 | 74 | 0.668 | 71 | 0.661 |
| **Lat_N1** | 211 | 0.097 | 79 | 0.839 | 76 | 0.448 |
| **Lat_N2** | 261 | 0.518 | 78 | 0.804 | 65 | 0.907 |
| **Lat_N3** | 346 | 0.298 | 66 | 0.442 | 37 | 0.144 |
| **Lat_REM** | 392 | 0.004 | 65 | 0.643 | 31 | 0.065 |
| **%N1** | 380 | 0.082 | 124 | 0.074 | 73 | 0.588 |
| **%N2** | 328 | 0.499 | 58 | 0.257 | 43 | 0.263 |
| **%N3** | 250 | 0.379 | 86 | 0.947 | 66 | 0.887 |
| **%REM** | 182 | 0.024 | 72 | 0.603 | 88 | 0.156 |
| **SE** | 241 | 0.289 | 61 | 0.318 | 59 | 0.842 |

**Table S2 - Pearson correlation with sleep architecture**

|  | r | CI95% | p-val |
| --- | --- | --- | --- |
| **TIB** | -0.07 | [-0.33 0.2 ] | 0.622 |
| **SPT** | -0.06 | [-0.32 0.21] | 0.648 |
| **WASO** | -0.14 | [-0.39 0.13] | 0.325 |
| **TST** | 0.09 | [-0.18 0.34] | 0.534 |
| **N1** | -0.03 | [-0.29 0.24] | 0.821 |
| **N2** | -0.05 | [-0.31 0.22] | 0.709 |
| **N3** | 0.18 | [-0.09 0.42] | 0.193 |
| **REM** | 0.18 | [-0.09 0.42] | 0.197 |
| **NREM** | 0.03 | [-0.23 0.3 ] | 0.808 |
| **SOL** | 0.02 | [-0.25 0.28] | 0.894 |
| **Lat_N1** | 0.06 | [-0.21 0.32] | 0.654 |
| **Lat_N2** | -0.05 | [-0.31 0.22] | 0.744 |
| **Lat_N3** | -0.31 | [-0.53 -0.05] | 0.021 |
| **Lat_REM** | -0.40 | [-0.61 -0.15] | 0.003 |
| **%N1** | -0.09 | [-0.35 0.18] | 0.519 |
| **%N2** | -0.11 | [-0.36 0.16] | 0.426 |
| **%N3** | 0.03 | [-0.24 0.29] | 0.843 |
| **%REM** | 0.24 | [-0.03 0.48] | 0.077 |
| **SE** | 0.24 | [-0.03 0.47] | 0.083 |

**Figure S2**

**
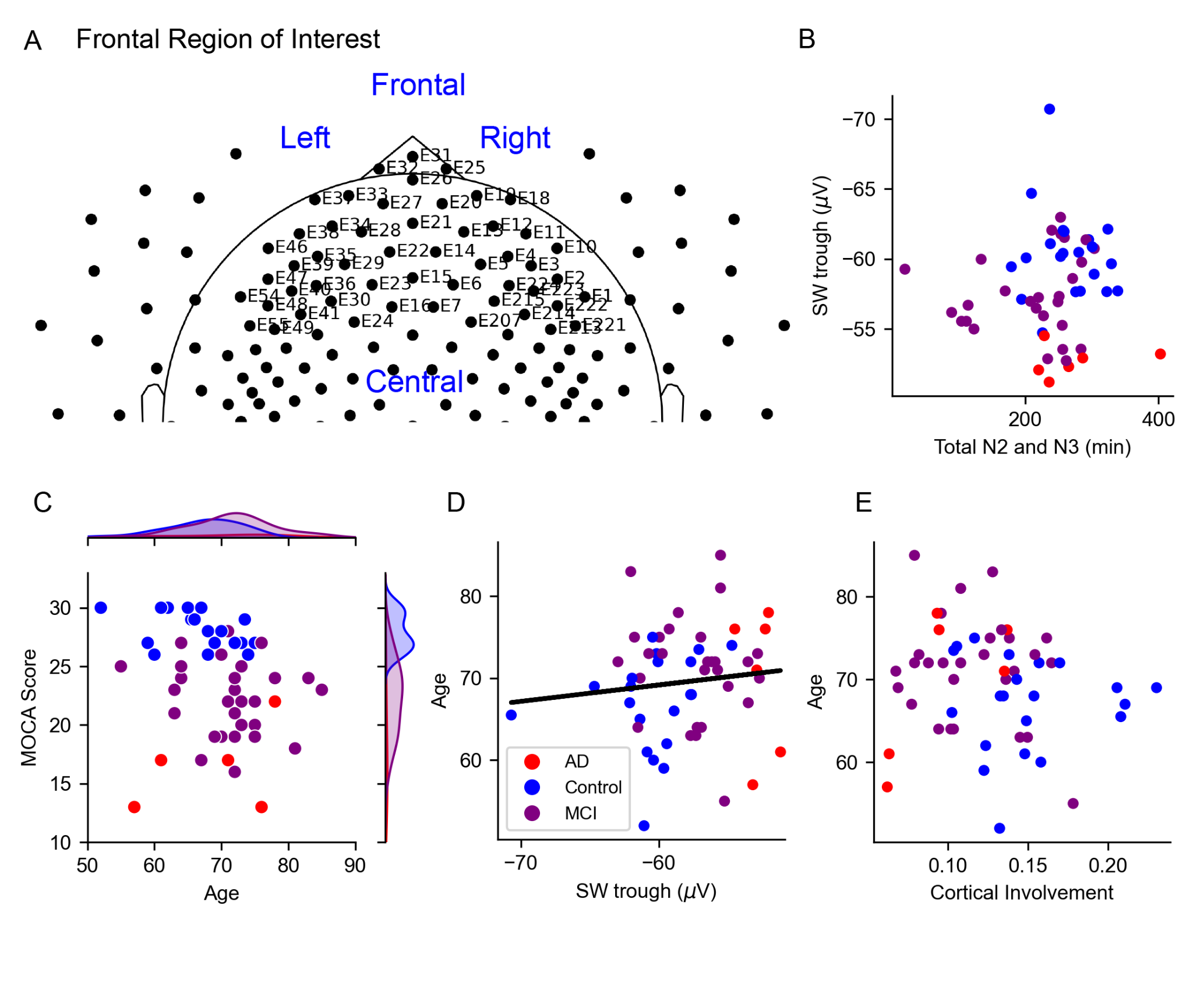
**

**(A)** Frontal Region of interest used for amplitude analysis according to EGI Magstim montage **(B)** Cohort was characterized by lack of correlation between age and MOCA score **(C)** Negative slow wave amplitude was not correlated to age the cohort **(D)** no relations between the slow-wave amplitude and NREM (N2+N3) data used for analysis.(E) No correlation between cortical involvement and age was observed.

**Figure S3**


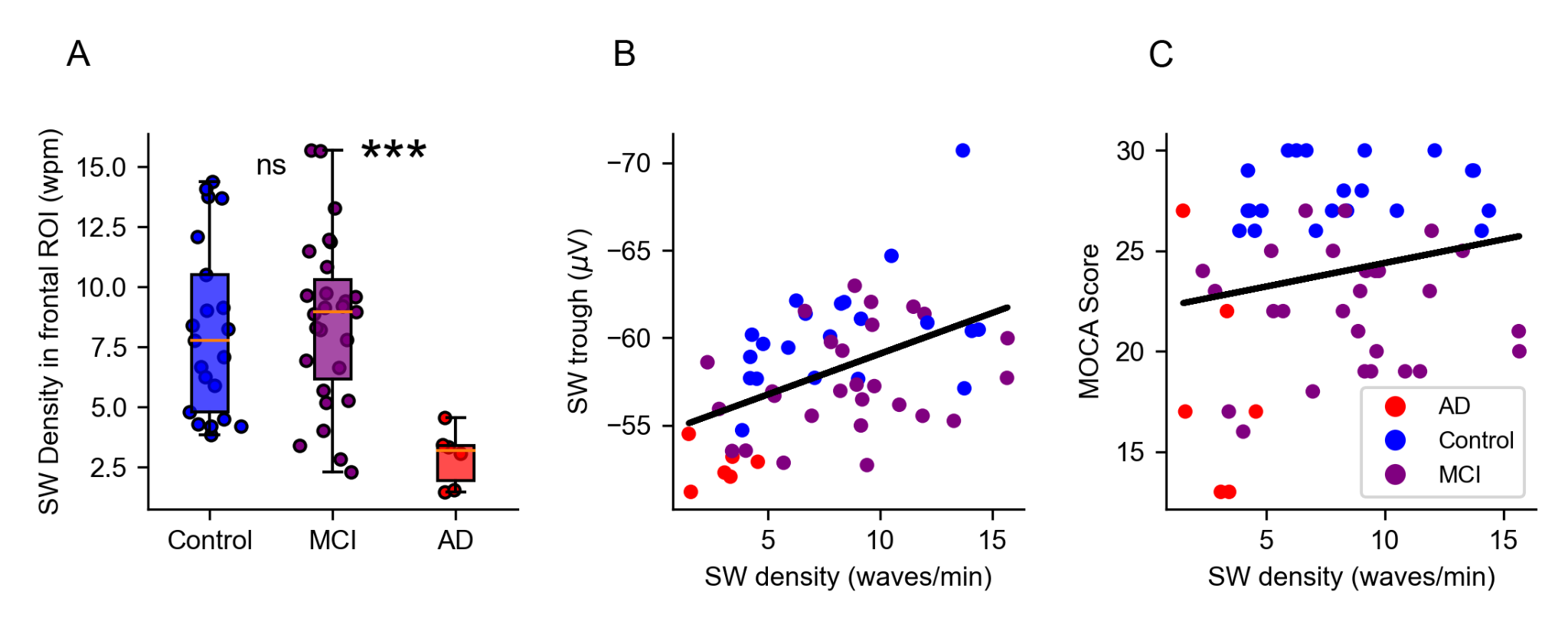


1. Comparison of Slow Wave (SW) density across groups. Healthy controls (blue) did not differ from aMCI patients (purple) had a similar average of 8.59±4.12 wpm (U=259, p=0.61), while AD patients (red) showed 2.91±1.43 wpm on average, significantly less compared with both aMCI patients (U=11, p=0.0003) and healthy controls (U=5, p=0.0001). While no significant difference where observed between MCI patients and Healthy controls, AD patients showed reduced SW density **(B)** SW density was significantly correlated to SW trough **(C)** MOCA score was not significantly correlated to SO density on the individual level.

**Data and code availability**

Data is available upon a reasonable request and in agreement with Tel Aviv University and Tel -Aviv Sourasky Medical Center data sharing policies. Custom Python code, beyond MNE, Yasa packages is available at:

<https://github.com/sharomer/eeg_2d_minimal_rejection>

Free open source sleep scoring software is available at:

<https://github.com/x64-bit/sleep-scoring/tree/main>
